# Sleep-wake functions and quality of life in patients with subthalamic deep brain stimulation for Parkinson’s disease

**DOI:** 10.1371/journal.pone.0190027

**Published:** 2017-12-18

**Authors:** Panagiotis Bargiotas, Lukas Eugster, Michael Oberholzer, Ines Debove, M. Lenard Lachenmayer, Johannes Mathis, Claudio Pollo, W. M. Michael Schüpbach, Claudio L. Bassetti

**Affiliations:** 1 Department of Neurology, University Hospital (Inselspital) and University of Bern, Bern, Switzerland; 2 Department of Neurosurgery, University Hospital (Inselspital) and University of Bern, Bern, Switzerland; National Institue on Drug Abuse, UNITED STATES

## Abstract

**Objectives:**

Sleep-wake disturbances (SWD) are frequent in Parkinson’s disease (PD). The effect of deep brain stimulation (DBS) on SWD is poorly known. In this study we examined the subjective and objective sleep-wake profile and the quality of life (QoL) of PD patients in the context of subthalamic DBS.

**Patients and methods:**

We retrospectively analyzed data from PD patients and candidates for DBS in the nucleus suthalamicus (STN). Pre-DBS, sleep-wake assessments included subjective and objective (polysomnography, vigilance tests and actigraphy) measures. Post-DBS, subjective measures were collected. QoL was assessed using the Parkinson’s Disease Questionnaire (PDQ-39) and the RAND SF-36-item Health Survey (RAND SF-36).

**Results:**

Data from 74 PD patients (62% male, mean age 62.2 years, SD = 8.9) with a mean UPDRS-III (OFF) of 34.2 (SD = 14.8) and 11.8 (SD = 4.5) years under PD treatment were analyzed. Pre-DBS, daytime sleepiness, apathy, fatigue and depressive symptoms were present in 49%, 34%, 38% and 25% of patients respectively but not always as co-occurring symptoms. Sleep-wake disturbances were significantly correlated with QoL scores. One year after STN DBS, motor signs, QoL and sleepiness improved but apathy worsened. Changes in QoL were associated with changes in sleepiness and apathy but baseline sleep-wake functions were not predictive of STN DBS outcome.

**Conclusion:**

In PD patients presenting for STN DBS, subjective and objective sleep-wake disturbances are common and have a negative impact on QoL before and after neurosurgery. Given the current preliminary evidence, prospective observational studies assessing subjective and objective sleep-wake variables prior to and after DBS are needed.

## Introduction

Sleep-wake disturbances (SWD) can appear in the early stages of Parkinson’s disease (PD), even as initial manifestations up to 10 years before motor symptoms onset[1]. As PD progresses, patients can experience a broad spectrum of SWD which affect wellbeing and daytime functioning[2].

Stimulation of the subthalamic nucleus (STN) improves Parkinsonian motor signs and quality of life (QoL)[3, 4]. Substantial effort has gone into studying factors that are associated with STN DBS outcome. Predictive factors for motor outcome and QoL after STN DBS are identified and have become inclusion criteria for STN DBS. These include the good response to levodopa[5], neurocognitive and psychological variables[6, 7], coordinate-based electrode location[8], patients’ baseline characteristics including age, disease duration[9, 10] and body mass[11]. For the QoL outcome after STN DBS, motor[12], neurocognitive and psychological variables[7] have been identified as predictive factors.

Sleep-wake functions in the context of STN DBS in patients with PD have not been extensively examined in the past[13] and their role as predictive factors for DBS outcome remains unclear. Recently, Baumann-Vogel et al. assessed subjective and objective (actigraphy and video-polysomnography) sleep-wake parameters in 50 PD patients prior to and 6 months after STN DBS. Both sleep quality (sleep continuity, sleep efficiency, percentage of deep sleep and time of accumulation of slow-wave activity) and daytime sleepiness improved after STN DBS. Rapid eye movement sleep features and fatigue were however refractory to subthalamic DBS[14]. Only few other studies used polysomnography to assess sleep prior to and after DBS surgery and most of them reported a positive impact of STN DBS on sleep architecture[15–20] (reviewed in Eugster et al.[13]). To the best of our knowledge no DBS study used vigilance tests to assess wake functions. Significantly more DBS studies used subjective measures to assess sleep-wake functions. After STN DBS subjective sleep quality is improved, the data on excessive daytime sleepiness are inconsistent, those for fatigue are limited and available data suggest worsening of apathy in PD patients after STN DBS [3, 15, 18, 21–35] (reviewed in Eugster et al.[13]).

In this study we used subjective and objective measures, including for the first time vigilance tests, to examine the sleep-wake profile and its associations, especially with the quality of life, of patients with PD presenting for STN DBS. In addition, we investigated the predictive value of baseline sleep-wake functions for motor and QoL outcome in PD patients treated with STN DBS.

## Methods

The protocol for this retrospective study was approved by the local ethics committee (2016–00369, Kantonale Ethikkommission Bern).

### Patients

We retrospectively analyzed data from subjects with PD[36] who underwent a sleep-wake assessment prior to STN DBS (inclusion and exclusion criteria for DBS as previously published[37]) at the University Hospital Bern between 2012 and 2016. The DBS surgery was performed as previously published[38]. The correct placement of the active contact within the STN was confirmed using MRI-based and electrophysiological results of microelectrode recording as previously described[39]. Briefly, direct postoperative stereotactic high-resolution head computer tomography (CT) scan was performed and superimposed onto a preoperative T2-weighted sequence to confirm leads and active electrode(s) locations. There was no lead location analysis showing active electrodes outside the STN and for none of the PD patients that underwent DBS surgery a revision of the electrode position was necessary. There were no sensorimotor or life-threatening complications in this group. In one subject, a small intraparenchymal bleed in the region of the left capsula interna and in a second subject a small intraclavicular haematoma encountered perioperatively. Both events were successfully managed postoperatively.

At baseline, motor signs, subjective and objective sleep-wake functions and QoL were assessed. The one-year postoperative follow-up assessment included motor signs, QoL and subjective sleep-wake measures.

### Motor assessment

The motor assessment included the modified Hoehn & Yahr (H&Y) stage and the Unified Parkinson’s Disease Rating Scale (UPDRS parts I-IV). The UPDRS was evaluated in “on” and “off” dopaminergic medication at baseline and at follow-up. Levodopa equivalent daily dose (LEDD) was calculated as previously described (including all commonly used medications in PD patients such as dopamine agonists, MAO-B inhibitors, COMT-inhibitors etc)[40].

### Subjective sleep-wake assessment

The Epworth Sleepiness Scale (ESS) provides a measurement of the patient’s subjective habitual level of daytime sleepiness, respectively how likely participants fall asleep in different situations. ESS is recommended for use in patients with PD[41]. It consists of 8 items with a 4-step scale[42]. Excessive daytime sleepiness is defined as an ESS score ≥10 (total score range 1–24).

Starkstein apathy scale (SAS) was used to assess apathy. SAS consists of 14 items phrased as questions that are to be answered by the patient on a four-point Likert scale. The total score ranges from 0 to 42, with higher scores indicating greater apathy. A SAS score of ≥14 is suggestive of significant apathy (total score range 0–42)[43].

The occurrence of fatigue was evaluated using the fatigue severity scale (FSS), a self-administered unidimensional generic 9-item fatigue rating scale. Although not explicitly recommended in the original study, a cut-off of ≥4 and a time frame covering the past 2 weeks are used by the developers and most other groups as evidence for an abnormal fatigue[44].

The 21-item version Hamilton depression rating scale (HAM-D) was used to evaluate depressive symptoms. For screening purposes in PD patients, a cut-off score of 9/10 has been suggested (total score range 0–54)[45].

### Objective sleep-wake assessment

#### Video-polysomnography

Standard nocturnal video-polysomnography (v-PSG) was performed during hospitalization as previously described[46]. The v-PSG consisted of six channel EEG (F3/M2, F4/M1, C3/M2, C4/M1, O1/M2, O2/M1), left and right electrooculography (EOG), submental, left and right anterior tibialis, flexor carpi radialis and adductor digiti minimi electromyography (EMG), electrocardiography (ECG), respiratory flow and effort, and pulse-oximetry. All recordings were performed by Embla RemLogic^™^ Software. Sleep stages and sleep-associated events were manually scored according to the American Academy of Sleep Medicine (AASM) scoring manual [47].

#### Actigraphy

Actigraphic ambulatory recordings were performed by the MotionWatch 8 actigraph (CamNtech Ltd., Cambridge, UK). The actigraph measures motor activity with a piezoelectric element. The patients were advised to wear the actigraph on the wrist of the non-dominant arm and recordings were performed for at least 7 days. Inactivity index (average amount of inactivity in 24h) was calculated as previously described[48].

#### Multiple sleep latency test (MSLT)

The MSLT measures the physiological tendency to fall asleep in the absence of alerting factors[49]. In our study, the MSLT was performed following an all-night v-PSG. During the test, electroencephalography, electrooculogram and electromyogram were recorded. Sleep latency and sleep staging were evaluated in 5 daytime nap opportunities (2 h apart) during the patient's daytime routine.

#### Maintenance of wakefulness test (MWT)

MWT is probably the most accurate measure of the ability to remain awake under soporific conditions for a defined period of time[50]. For the MWT, patients were asked to stay awake in a quiet, dark room during 4 wakefulness opportunities (2 h apart) while sitting in a recliner. The duration of each test was 40 minutes at maximum or until 3 consecutive epochs of sleep were scored.

### QoL assessment

For the assessment of QoL we used the Parkinson’s Disease Questionnaire (PDQ-39) and the RAND SF-36-item Health Survey (RAND SF-36), both recommended for use in PD[51].

PDQ-39 consists of 39 questions, covering eight domains that are combined in a summary index where 0 denotes "best QoL" and 100 "worst QoL"[52].

RAND SF-36 is a standardized questionnaire and one of the most widely used of the health-related quality of life measures[53]. The questionnaire consists of 36 questions/items measuring physical health composite (PHC) and mental health composite (MHC) in relation to eight health concepts. Higher scores represent better self-perceived health[53].

### Statistical analysis

The mean values at baseline and post-DBS were compared using a *t* test for paired samples, after being tested for normal distribution using the Kolmogorov-Smirnov test. Bivariate correlation analysis was performed to identify variables that were related to motor and QoL scores at baseline and post-DBS and to identify predictors for the DBS outcome. Changes in UPDRS-III, LEDD, ESS, SAS, PDQ-39 and RAND SF-36 were correlated with the following baseline parameters using Pearson’s correlation: Age, gender, years under PD medication, HAM-D total score, baseline motor and non-motor scores (UPDRS I-IV), levodopa equivalent daily dose (LEDD), SAS score, ESS score, FSS score, inactivity index, sleep latency in MSLT and in MWT and several PSG parameters. Variables with the values of p <0.05 were also included in a multiple linear regression analysis. Two-tailed statistical significance level was set at p <0.05.

Statistical analysis was performed on SPSS-18 and Prism 7 for Windows (GraphPad Software, Inc., San Diego, CA, USA).

## Results

Data from 88 subjects with PD were collected. Fourteen subjects were excluded from further analysis. Eleven of them due to high amount of missing data and three because of previous DBS treatment in other targets. We included 74 subjects in the study. Objective measures were available only preoperatively. The n number of included subjects for each subjective and objective assessment is presented in the tables of the manuscript. Baseline demographic and disease characteristics and most importantly the extent of motor and QoL improvement in our cohort were comparable with those published in other large prospective, multicenter observational cohort DBS studies[4]. Before surgery, the mean age of the sample was 62.2 years with a mean of 11.8 years under PD medication, 38% were female. The mean preoperative motor subscore was 34.2 and mean preoperative LEDD was 1069mg. Table 1 presents preoperative baseline characteristics for the entire cohort.

**Table 1:** Baseline demographic and clinical characteristics of the patients (n = 74).

	Mean (± SD)
**Mean age (in years)**	62.2 ± 8.9
**Women (%)**	38
**Years of PD medication**	11.8 ± 4.5
**UPDRS-I**	1.8 ± 1.9
**UPDRS-II (OFF)**	15.5 ± 6.5
**UPDRS-III (OFF)**	34.2 ± 14.8
**H&Y (OFF)**	2.7 ± 0.9
**MMSE**	28.6 ± 1.3
**LEDD (in mg)**	1069.0 ± 658.3

H&Y, Hoehn and Yahr; UPDRS, unified parkinson’s disease rating scale; MMSE, Mini-Mental State Examination; LEDD, levodopa equivalent daily dose.

### Sleep-wake profile of PD patients presenting for STN DBS

**Fig 1:**
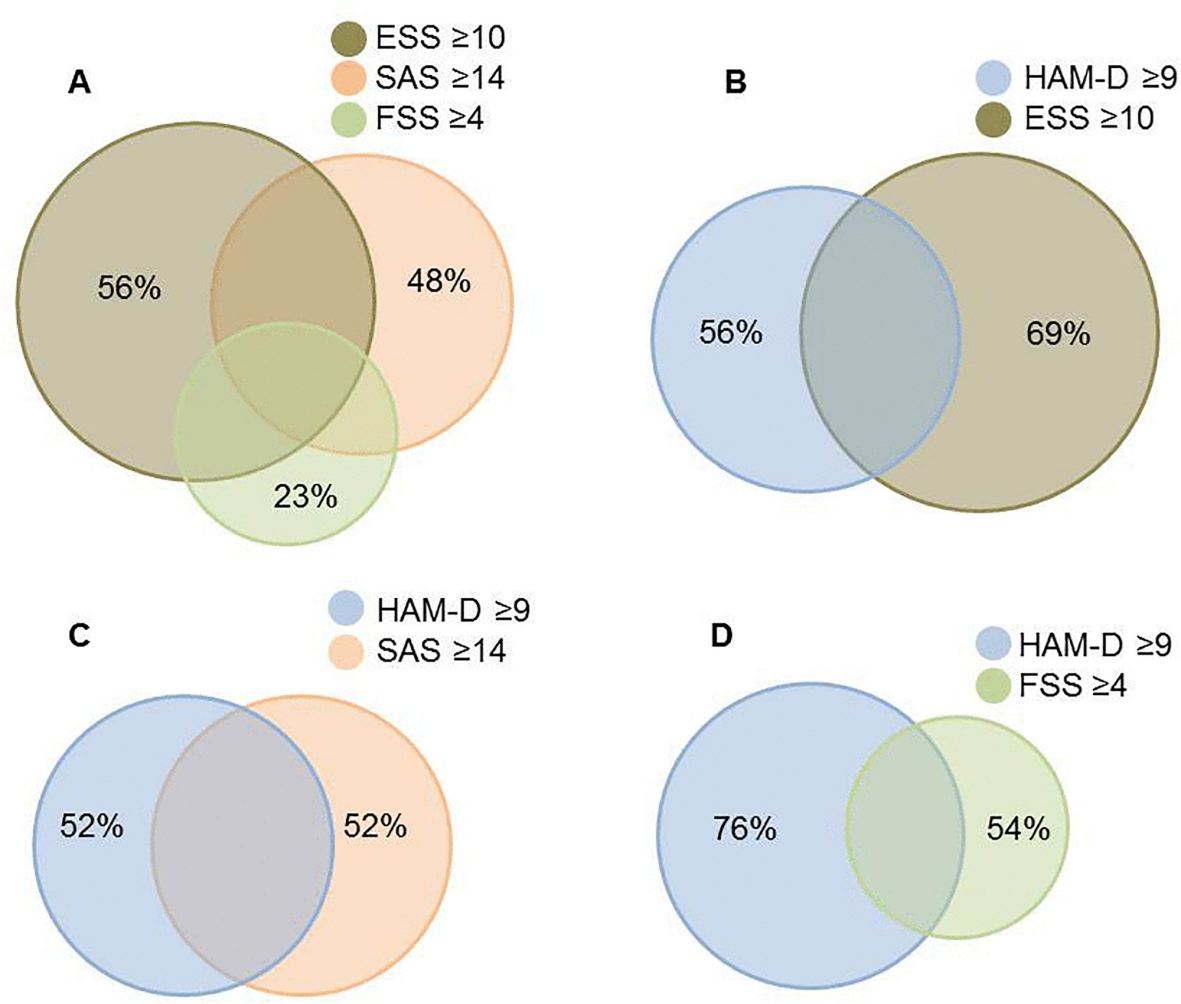
Venn diagram of proportions and overlaps of symptoms in PD patients presenting for STN DBS. At baseline, overlap between sleepiness, fatigue and apathy was common but a significant percentage of patients report “pure” symptoms without concomitant comorbidities (A). Distinction and overlap of sleepiness (B), apathy (C) and fatigue (D) with depressive symptoms. The area of a circle is proportional to the number of observed individuals. The percentage of subjects without overlaping symptoms in each subgroup is shown. HAM-D, Hamilton depression rating scale (21 Items); SAS, Starkstein apathy scale; FSS, fatigue severity scale; ESS, Epworth sleepiness scale.

At baseline prior to STN DBS, subjective daytime sleepiness (ESS ≥10), apathy (SAS ≥14) and fatigue (FSS ≥4) were present in 49%, 34% and 38% of patients, respectively. Increased scores of the depression rating scale (HAM-D ≥9) were found in 25% of the patients. A bivariate analysis failed to provide statistically significant correlations between sleepiness, fatigue, apathy and depression scores (data not shown). Despite considerable overlap, daytime sleepiness, apathy and fatigue did not always co-occur (Fig 1A). In addition, our data showed that 69% of patients with sleepiness, 52% of patients with apathy, and 54% of patients with fatigue reported no significant depressive symptoms (Fig 1B, 1C and 1D).

In MSLT, 25 out of 36 (69%) had a mean sleep latency below 10 minutes and 13 subjects (36%) had a mean sleep latency below 5 minutes. In MWT, only one subject had a sleep latency lower than 10 minutes[54]. In v-PSG, half of the patients had a sleep efficiency <80%, and one third <70%. Moreover, sleep architecture was disturbed as shown by increased percentage of stage 1 sleep and decreased rapid eye movement (REM) sleep compared to the age dependent normal values[55] (although in the summary from Ohayon et al. most of the included PSG studies in adults have used the Rechtschaffen and Kales scoring rules[56](Table 2). Twenty-two out of 50 subjects (44%) showed polysomnographic and clinical features of REM sleep Behavioral Disorder (RBD), 11 out of 50 subjects (22%) had an apnea-hypopnea-index (AHI) ≥15/h, and 11 out of 50 subjects (22%) had a PLM (periodic limb movements)-index ≥15/h. In actigraphy, 8 out of 34 subjects (21%) had an inactivity index ≥35%. Table 2 shows the mean values for several objective sleep-wake parameters at baseline.

**Table 2:** Baseline objective sleep-wake parameters.

	n	Mean (± SD)
Sleep efficiency (%)*	50	74.9 ± 15.0
**REM sleep (% of total sleep)**	50	15.4 ± 10.1
**Stage 1 sleep (% of total sleep)**	50	19.7 ± 12.3
**Stage 2 sleep (% of total sleep)**	50	51.6 ± 13.4
**Slow wave sleep (% of total sleep)**	50	13.2 ± 11.1
Wake after sleep onset (%)*	50	25.5 ± 15.2
**Apnea-hypopnea index (/h)**	50	11.0 ± 15.2
**Periodic limb movements index (/h)**	50	12.7 ± 22.9
**Inactivity index (% per 24 hours)**	34	28.0 ± 8.7
**Sleep latency in MSLT (in min)**	36	8.7 ± 5.7
**Sleep latency in MWT (in min)**	30	29.9 ± 11.3

Preoperative polysomnography was performed in 50 patients, preoperative MSLT in 36 patients, preoperative actigraphy in 34 patients and preoperative MWT in 30 patients.

SD, standard deviation; REM, rapid eye movement; MSLT, multiple sleep latency test; MWT, maintenance of wakefulness test

*relative to sleep period time

At baseline, we found no significant correlation between subjective and objective sleep-wake variables (data not shown). Finally, higher LEDD and higher scores in UPDRS and H&Y scales were not associated with worse sleep-wake function (data not shown).

### Sleep-wake profile and QoL in PD patients presenting for STN DBS

At baseline, sleep efficiency was negatively and scores in ESS, SAS and FSS were positively correlated with PDQ-39 summary index and several PDQ-39 dimensions. Scores in SAS and FSS were negatively correlated with SF-36 RAND physical health composite (PHC) und mental health composite (MHC) and several SF-36 dimensions (Table 3 and S1 Table). These relationships remained significant even after adjustment for PD stage, motor score and LEDD (data not shown).

**Table 3:** Pearson correlations of baseline sleep-wake scores with QoL measures.

	RAND SF-36		PDQ-39
	PHC	MHC	SI
**SAS**	-0.20	-0.39**	0.27*
**FSS**	-0.37*	-0.43*	0.36*
**ESS**	-0.21	-0.19	0.24*
**Seff**	-0.05	0.12	-0.38**

SAS, Starkstein apathy scale; FSS; fatigue severity scale; ESS, Epworth sleepiness scale; Seff, sleep efficiency; RAND SF-36, RAND SF-36-item Health Survey; PDQ-39, Parkinson’s disease questionnaire; PHC, physical health composite; MHC, mental health composite; SI, summary index. The numbers represent r values. Values below zero express negative correlation.

*p values <0.05 and

**p values <0.01 (two tailed) were regarded as significant.

### Evolution of sleep-wake profile and QoL after STN DBS

**Fig 2:**
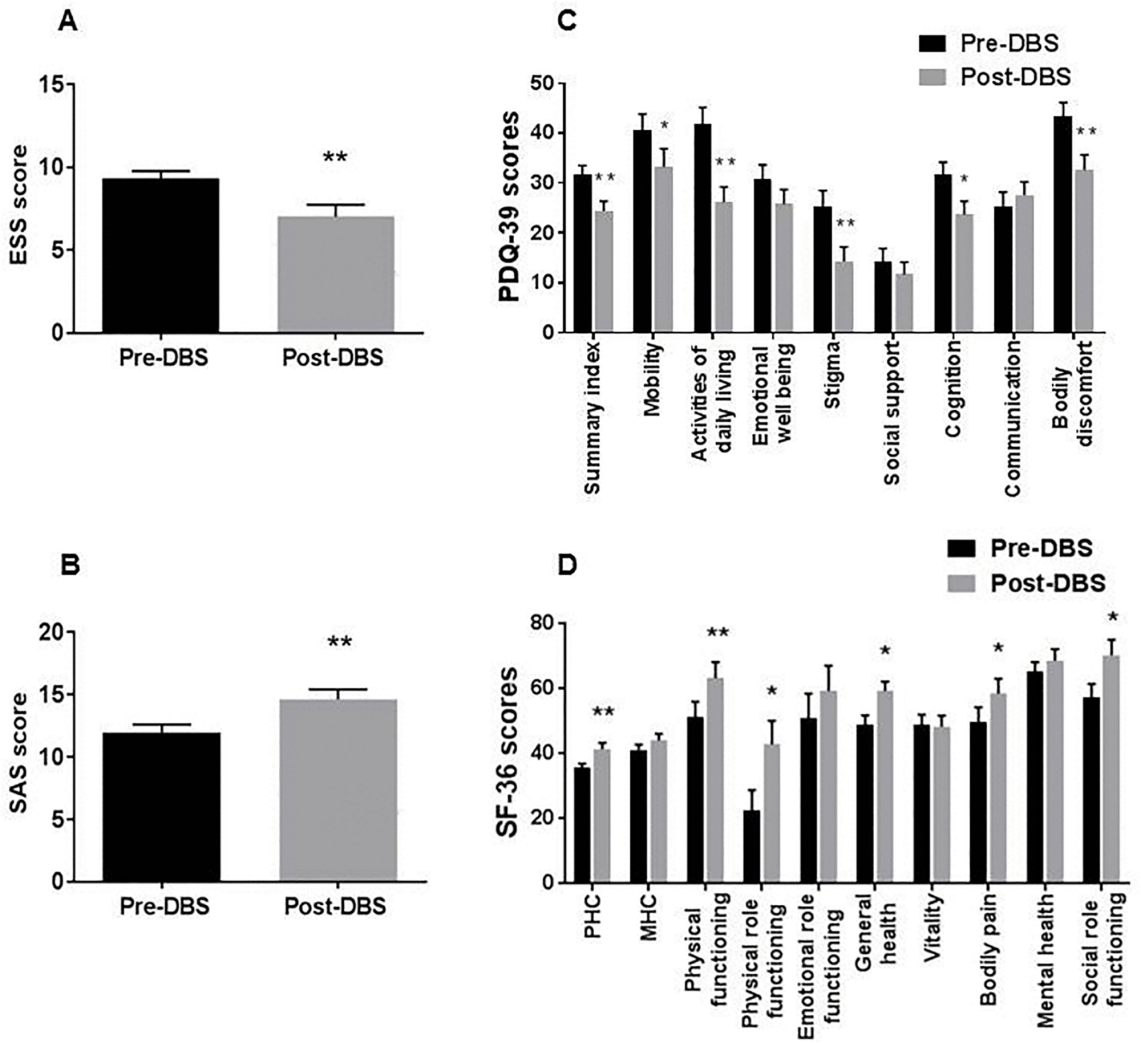
Post-DBS change in sleepiness, apathy and QoL scores. One year after STN DBS, (A) ESS mean score was significantly decreased and (B) SAS mean score was significantly increased in relation to the baseline (n = 43–46). PDQ-39 summary index and several dimensions in PDQ-39 (C) and in RAND SF-36 (D) improved significantly in comparison to baseline (n = 32 and 45 respectively); paired t-test *p <0.05 and **p <0.01). Error bars represent the standard error of the mean, SEM. DBS, deep brain stimulation; QoL, quality of life; PDQ-39, Parkinson’s disease questionnaire; RAND SF-36, RAND SF-36-item Health Survey; PHC, physical health composite; MHC, mental health composite.

One year after STN DBS, motor score (UPDRS-III, n = 46) was significantly improved by 46% (post-DBS, on stimulation without medication vs. pre-DBS without medication) and the mean LEDD (n = 46) was reduced by 61%. Scores in cognitive scale remained unchanged (mean MMSE 28.7±1.7 compared to 28.6±1.3 at baseline). Sleepiness score improved and apathy score increased (Fig 2A and 2B). The PDQ-39 showed a 23% improvement of the summary index (SI) and a significant improvement in several PDQ-39 domains, including mobility, activities of daily living, social stigma, cognition, and bodily discomfort. In RAND SF-36, improvements were shown in PHC and in physical functioning, physical and social role functioning, bodily pain and general health perception. The results of QoL scores are summarized in Fig 2(C) and 2(D).

After adjustment for motor scores and LEDD, change in SAS score was associated with changes in PDQ-39 summary index and SF-36 RAND MHC and PHC and change in ESS score was positively associated with PDQ-39 summary (Table 4).

**Table 4:** Association of post-DBS changes in QoL with change scores of several progression variables.

	*Δ*PDQ-39 SI			*Δ*SF-36_MHC			*Δ*SF-36_PHC		
	β^a^	SE β	β	β^a^	SE β	β	β^a^	SE β	β
***Δ*SAS**	0.97	0.35	0.43*	-0.83	0.38	-0.45*	-0.53	0.33	-0.34*
***Δ*ESS**	1.12	0.48	0.39*	-0.15	0.55	-0.06	-0.04	0.47	-0.02
***Δ*UPDRS-III (OFF)**	0.20	0.17	0.21	-0.06	0.30	-0.05	-0.18	0.26	-0.20
***Δ*LEDD**	-0.00	0.00	-0.20	0.00	0.00	0.08	-0.00	0.00	-0.10

UPDRS, unified parkinson’s disease rating scale; LEDD, levodopa equivalent daily dose; PDQ-39, Parkinson’s disease questionnaire; SAS, Starkstein apathy scale; ESS, Epworth sleepiness scale; Δ, represents change of a quantity; SE standard error.

*p values <0.05 (two tailed) were regarded as significant.

^a^Unstandardized beta coefficients.

### Predictive value of sleep-wake functions for STN DBS outcome

Significant interactions were found between baseline ESS and SAS scores and changes in ESS and SAS respectively. These interactions remained significant even after adjustment for motor scores and LEDD (Table 5). However, we found no significant relationship between baseline sleep-wake test results and changes in motor and QoL ratings after STN DBS (Table 6).

**Table 5:** Association of baseline variables with post-DBS changes in sleepiness and apathy score.

	*Δ*ESS			*Δ*SAS		
	β^a^	SE β	β	β^a^	SE β	β
**SAS**	0.03	0.12	0.03	0.50	0.12	0.53**
**ESS**	0.33	0.16	0.32*	-0.01	0.19	-0.01
**UPDRS-III (OFF)**	-0.01	0.04	-0.02	-0.01	0.05	-0.04
**LEDD**	0.00	0.00	0.13	0.00	0.00	0.12

LEDD, levodopa equivalent daily dose; UPDRS, unified parkinson’s disease rating scale; ESS, Epworth sleepiness scale; Δ, change; SE standard error.

*p values <0.05 and

**p values <0.01 (two tailed) were regarded as significant.

^a^Unstandardized beta coefficients.

**Table 6:** Association of baseline sleep-wake variables with DBS motor and QoL outcome.

	*Δ*PDQ-39		*Δ*SF-36_MHC		*Δ*SF-36_PHC		*Δ*UPDRS-III	
	Pearson r	p	Pearson r	p	Pearson r	p	Pearson r	p
**ESS**	0.03	0.82	-0.01	0.97	0.31	0.08	-0.09	0.60
**SAS**	0.24	0.10	-0.25	0.16	-0.19	0.27	-0.17	0.30
**FSS**	0.26	0.32	-0.09	0.86	-0.49	0.26	0.04	0.90
**Seff**	-0.24	0.22	0.11	0.67	-0.15	0.55	-0.06	0.80

PDQ-39, Parkinson’s disease questionnaire; RAND SF-36, RAND SF-36-item Health Survey; UPDRS, unified parkinson’s disease rating scale; ESS, Epworth sleepiness scale; SAS, Starkstein apathy scale; FSS, fatigue severity scale; Seff, sleep efficiency; SL, sleep latency, Δ represents change of a quantity.

## Discussion

In this study we used subjective and objective measures to assess the sleep-wake profile of candidates for STN DBS for PD and to investigate its relationship with quality of life in the context of a DBS surgery.

Our results showed that, in PD patients presenting for STN DBS, wake disturbances were frequent and sleep quality was poor. Despite considerable overlap, subjective daytime sleepiness, fatigue, apathy, and depression were distinct and occurred also as “pure” symptom, i.e. isolated. These findings further support the notion that SWD do not occur in PD solely as part of other non-motor symptoms (e.g. depression) or as side-effects of dopaminergic medication but may represent manifestations of PD that may occur even in the drug-naïve phase worsen with disease progression. The short sleep latency in MSLT was suggestive for severe excessive daytime sleepiness whereas the scores in MWT and in the actigraphy were consistent with preserved ability to remain alert and physical daytime activity. However, it cannot be excluded that an increased physical inactivity during the day could be masked by the increased severity in disease symptoms (e.g dyskinesias) in these patients, which cannot be distinguished by actigraphy. The main polysomnographic findings can be summarized as followings: the disrupted sleep macroarchitecture, the reduced sleep efficiency and in about one fifth of the patients the presence of PLM in sleep and the disturbed respiration suggestive for the presence of at least a mild sleep-associated breathing disorder. Interestingly, the prevalence and severity of SWD were independent of the disease and motor disability.

In recent years, interest in QoL and its associated factors in the context of DBS progressively increased[57–59]. QoL often improves after STN DBS, but not in all PD patients, and this despite the improvement in motor scores and reduction in dopaminergic medication[60]. In our cohort, severe SWD at baseline reflected poor QoL, especially in dimensions related to cognitive and emotional function, vitality and mental health. The association between SWD and QoL was independent of disease stage and motor disability. One year after STN DBS, motor scores and QoL improved. Interestingly, changes in QoL were stronger correlated with changes in sleepiness and apathy and less with changes in motor scores and dopaminergic medication. This emphasizes the importance of these non-motor aspects for QoL in patients with PD[57].

Our findings of increased apathy and improved sleepiness after STN DBS are consistent with the majority of findings in the available literature (reviewed in Eugster et al[13]). Interestingly, changes in sleepiness and apathy after STN DBS were associated with pre-surgical sleepiness and apathy scores but not with the reduction of the dopaminergic medication or the improvement of motor signs. This further supports the notion that non-dopaminergic networks are crucial for both symptoms in PD. Finally, no baseline sleep-wake subjective or objective variable was associated with motor and QoL scores after STN DBS, suggesting that SWD cannot predict STN DBS outcome.

SWD in PD are multifactorial and although essential the assessment of all possible factors that contribute to their occurrence or worsening (e.g medication) was outside the scope of the current study. In addition, the retrospective design of this study and the long-term follow-up (12 months) do not allow us to assess the confounding effects of the disease progression on sleep-wake functions and disentangle them from the impact of STN DBS itself. This together with the lack of postoperative objective sleep-wake measures and the different numbers of included subjects in each assessment consist important limitations of the current study.

## Conclusions

Our findings underline the high prevalence of SWD and their strong association with QoL in candidates for STN DBS. The inclusion of sleep-wake assessements may not only advance pre-DBS selection procedures but will contribute to the improvement of the therapeutic efforts following DBS. Given the current preliminary evidence, prospective observational DBS studies assessing subjective and objective sleep-wake variables prior to and after DBS are needed to further elucidate the impact of medication, DBS and PD itself on sleep and wakefulness.

## Supporting information

S1 Table Pearson correlations of QoL with baseline sleep-wake functions scores. At baseline, sleep efficiency in polysomnography was negatively and scores for apathy, fatigue and sleepiness were positively correlated with several PDQ-39 dimensions. In addition, sleep efficiency in polysomnography was positively and scores for apathy (SAS), fatigue (FSS) and sleepiness (ESS) were negatively correlated with several SF-36 dimensions. SAS, Starkstein apathy scale; FSS; fatigue severity scale; ESS, Epworht sleepiness scale; Seff, sleep efficiency; n, number; RAND SF-36, short form (36) health survey, PDQ-39, Parkinson’s disease questionnaire. PF, physical functioning; RP, physical role functioning; RE, emotional role functioning; GH, general health; VT, vitality, BP, bodily pain, MH, mental health; SF, social role functioning; MOB, mobility; ADL, activities of daily living; EWB, emotional well-being; SS, social stigma; SoSu, social support; COG, cognition, COM, communication; BP, bodily discomfort. The numbers represent r values. Values below zero express negative correlation. *p values < 0.05 and **p values < 0.01 (two tailed) were regarded as significant.
(DOCX)
