# Supplementary material for: Sleep-wake functions and quality of life in patients with subthalamic deep brain stimulation for Parkinson’s disease
Source: PLoS One. 2017 Dec 18;12(12):e0190027. doi: 10.1371/journal.pone.0190027 (PMC5734707; doi:10.1371/journal.pone.0190027)
Supplement: S1 Table — At baseline, sleep efficiency in polysomnography was negatively and scores for apathy, fatigue and sleepiness were positively correlated with several PDQ-39 dimensions. In addition, sleep efficiency in polysomnography was positively and scores for apathy (SAS), fatigue (FSS) and sleepiness (ESS) were negatively correlated with several SF-36 dimensions. SAS, Starkstein apathy scale; FSS; fatigue severity scale; ESS, Epworht sleepiness scale; Seff, sleep efficiency; n, number; RAND SF-36, short form (36) health survey, PDQ-39, Parkinson’s disease questionnaire. PF, physical functioning; RP, physical role functioning; RE, emotional role functioning; GH, general health; VT, vitality, BP, bodily pain, MH, mental health; SF, social role functioning; MOB, mobility; ADL, activities of daily living; EWB, emotional well-being; SS, social stigma; SoSu, social support; COG, cognition, COM, communication; BP, bodily discomfort. The numbers represent r values. Values below zero express negative correlation. *p values < 0.05 and **p values < 0.01 (two tailed) were regarded as significant. (DOCX) [file pone.0190027.s001.docx]

|  | **RAND SF-36** | | | | | | | | **PDQ-39** | | | | | | | |
| --- | --- | --- | --- | --- | --- | --- | --- | --- | --- | --- | --- | --- | --- | --- | --- | --- |
|  | PF | RP | RE | GH | VT | BP | MH | SF | MOB | ADL | EWB | SS | SoSu | COG | COM | BD |
| **SAS** | -0.34^**^ | -0.08 | -0.15 | -0.10 | -0.40^**^ | -0.02 | -0.39^**^ | -0.25^*^ | 0.06 | 0.18 | 0.28^*^ | 0.21 | 0.17 | 0.24^*^ | 0.30^*^ | -0.02 |
| **FSS** | -0.26 | -0.11 | -0.25 | -0.39^*^ | -0.52^**^ | -0.29 | -0.39^*^ | -0.17 | 0.26 | 0.02 | 0.35^*^ | 0.30 | 0.09 | 0.44^*^ | 0.26 | 0.16 |
| **ESS** | -0.17 | -0.16 | -0.19 | 0.09 | -0.12 | -0.25^*^ | -0.09 | -0.19 | 0.08 | -0.06 | 0.03 | 0.03 | 0.25^*^ | 0.44^**^ | 0.03 | 0.12 |
| **Seff** | -0.00 | -0.22 | 0.01 | -0.13 | 0.32^*^ | 0.25 | 0.23 | -0.23 | -0.12 | -0.25 | -0.31^*^ | -0.14 | -0.14 | -0.40^**^ | -0.34^*^ | -0.22 |

**S1 Table** Pearson correlations of QoL with baseline sleep-wake functions scores.
